# Comprehensive Snake Venomics of the Okinawa Habu Pit Viper, *Protobothrops flavoviridis*, by Complementary Mass Spectrometry-Guided Approaches

**DOI:** 10.3390/molecules23081893

**Published:** 2018-07-29

**Authors:** Maik Damm, Benjamin-Florian Hempel, Ayse Nalbantsoy, Roderich D. Süssmuth

**Affiliations:** 1 Institut für Chemie, Technische Universität Berlin, 10623 Berlin, Germany; maik.damm@tu-berlin.de (M.D.); benjamin.hempel@chem.tu-berlin.de (B.-F.H.); 2 Department of Bioengineering, Ege University, 35100 Izmir, Turkey; analbantsoy@gmail.com

**Keywords:** snake venomics, viperidae, *Protobothrops flavoviridis*, Habu pit viper, bottom-up, top-down, BPP, tripeptide metalloprotease inhibitor, cytotoxicity

## Abstract

The Asian world is home to a multitude of venomous and dangerous snakes, which are used to induce various medical effects in the preparation of traditional snake tinctures and alcoholics, like the Japanese snake wine, named Habushu. The aim of this work was to perform the first quantitative proteomic analysis of the *Protobothrops flavoviridis* pit viper venom. Accordingly, the venom was analyzed by complimentary bottom-up and top-down mass spectrometry techniques. The mass spectrometry-based snake venomics approach revealed that more than half of the venom is composed of different phospholipases A2 (PLA_2_). The combination of this approach and an intact mass profiling led to the identification of the three main Habu PLA_2_s. Furthermore, nearly one-third of the total venom consists of snake venom metalloproteinases and disintegrins, and several minor represented toxin families were detected: C-type lectin-like proteins (CTL), cysteine-rich secretory proteins (CRISP), snake venom serine proteases (svSP), l-amino acid oxidases (LAAO), phosphodiesterase (PDE) and 5′-nucleotidase. Finally, the venom of *P. flavoviridis* contains certain bradykinin-potentiating peptides and related peptides, like the svMP inhibitors, pEKW, pEQW, pEEW and pENW. In preliminary MTT cytotoxicity assays, the highest cancerous-cytotoxicity of crude venom was measured against human neuroblastoma SH-SY5Y cells and shows disintegrin-like effects in some fractions.

## 1. Introduction

Since ancient times, people have been fascinated by snakes and ascribed to them a diverse set of properties and character traits. Especially, in mythologies from South America through ancient Egypt to the Asian world, snakes and snake-like creatures represent both good and evil [1,2,3,4]. Most of them appear as symbols of wisdom and protection, and healing aspects have often been attributed to them [2]. Even today, the Aesculapius, the snake-wrapped rod of the Hellenic god Asclepius, and the winged Caduceus of Hermes symbolize medical, veterinarian and pharmacological professions [3]. On the other hand, based on encounters with humans, snakes are also known and feared for their bites and their possible consequences. Most accidents with snakes were registered in the tropics and mainly involve contact with farmers during field work. In consequence, snake envenomation was restored, in 2017, to the list of neglected tropical diseases [5].

One of the perillous regions is Japan, home to more than 38 different venomous and non-venomous snakes [6]. They coexist with man over the entire country, even on the smaller islands, wherein the diverse climatic conditions play a decisive role in the distribution of snakes [7]. The more densely inhabited areas exhibit a higher risk of bites and envenomation, which is further increased in the subtropical regions during the warm summer months [7]. Representatives of the most dangerous snake families in Japan are the Habu (*Protobothrops flavoviridis*; *P. flavoviridis*) and Mamushi (*Gloydius blomhoffii*) pit vipers, as well as the colubrid, Yamakagashi (*Rhabdophis tigrinus*) and the oceanic Erabu Umi Hebi (*Laticauda semifasciata*) [7,8,9].

Particularly in several Asian countries, but also in other countries, many snakes, mostly dangerous ones, were used in special traditional medicines, e.g., in Japan, the *P. flavoviridis*, also known as the Okinawa Habu, which gave the Habushu snake wine its name, because it contains a snake or snake extract [10,11]. Commonly, these liquors contain high ethanol concentrations of ~40% and are unproblematic to consume. However, in home-made liquors, lower ethanol concentrations are also feasible, so that, in rare cases, drinking can cause intoxications as well as health impairment or damage [12].

The Japanese Habu viper is a member of the venomous pit viper subfamily. In some cases, *P. flavoviridis* is classified as another genus and named, *Trimeresurus flavoviridis* [13]. This snake is endemic to 25 islands between Japan and Taiwan, whereas the largest areas of distribution are the Amami (Kagoshima Prefecture) and the Okinawa Islands (Okinawa Prefecture). Morphological and genetic differences in these populations are still being studied, in which a genetic gap between the two island populations was shown [14,15]. The Habu is responsible for most envenomations on the Amami Ohshima islands and nearby regions [16]. A bite, like that of one of the other vipers, could lead to less dangerous, more local symptoms (pain, erythema, vomiting), or to more dramatic symptoms, such as necrosis, acute kidney injury and death. At the beginning of the 20th century, the mortality rate was 10% [16,17]. The enhanced medical supply chains helped to decrease mortality to nearly 1% in 2013 [16]. One further main reason for the lower mortality rate is the development of potent antivenoms, which were, until now, the only effective treatment for snake envenomation [18]. Since the start of antivenom serotherapy trials against snake bites, more than 120 years have elapsed, but, until the present day, snakebites are still a serious threat to humans, particularly those working in endemic areas [5,19]. The composition of venoms defines the effect of an envenomation and is relevant for the development of specific and effective antivenoms, but it also could provide components with physiological functions, exploited for the development of drugs [20]. Hence, apart from the investigation of snake venoms for providing therapies and cures for snake bites, the exploration of their drug potential represents an important aspect of venom research.

As already mentioned above, venoms are a complex mixture of low molecular weight substances, e.g., nucleotides, sugars and lipids, as well as different peptides and proteins, the latter of which has enzymatic and non-enzymatic functions [18,21,22,23]. Habitat variation and the divergence of prey, which exert an evolutionary pressure, are major aspects that could lead to changes in the venom composition [14,24,25]. Even if the percentage composition differs from species to species, and also between different separated populations of the same species, the main toxin families in snakes have mostly been identified [18,26]. The toxins of vipers range from small natriuretic peptides, bradykinin-potentiating peptides (BPPs), over cysteine-rich secretory proteins (CRISPs) and phospholipases A2 (PLA_2_s) to high molecular serine- and metalloproteinases (svSPs, svMPs) [25,27].

The venom of *P. flavoviridis* has been under investigation for decades and is still a source of new findings, e.g., in the case of C-type lectin-like proteins (CTL), which are known for their strong effect on platelet aggregation [28,29,30,31,32,33]. Hence, two CTLs were firstly identified in the Habu snake venom. One CTL binds the blood coagulation factors, IX and X (named IX/X-BP), whereas the second has been described as an antagonist of the Willebrand factor receptor, GPIb, and is called, flavocetin-A [34,35]. Recent studies have shown that flavocetin-A, as a well-known protein, also inhibits the collagen-binding α2β1 integrin, which is the main receptor of platelets and necessary for platelet and cell activation [33,36].

Previous studies on the venom of *P. flavoviridis* were transcriptomic approaches and proteomic studies, yet limited to a shot-gun analysis, which could not provide a detailed picture of the venom composition [31,37]. Here, we report the first quantitative analysis of the *P. flavoviridis* venom, by a combination of diverse mass spectrometric methods, to give a more accurate profile of the venom and its composition. Therefore, a combined proteomic approach of bottom-up (BU) and top-down (TD) mass spectrometry (MS), including intact mass profiling (IMP), was used for snake venomic analyses, thus ensuring a high annotation coverage of the whole venom [38,39,40].

## 2. Results

### 2.1. Top-Down Analysis

**Figure 1:**
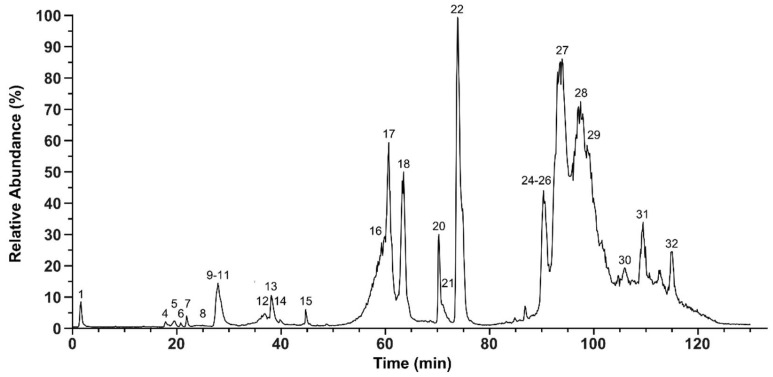
Total ion chromatogram of the *P. flavoviridis* venom for IMP and TD. The total ion counts in *P. flavoviridis* crude venom were measured by the HPLC-ESI-MS of native crude venom. The relative abundance was set to 100% for the highest peak. The peak nomenclature is based on the chromatogram fractions (shown in Figure 2). The identified molecular masses of intact peptides and proteins are listed Table S1.

In a first top-down (TD) analytical run, the venom of *P. flavoviridis* was analyzed by a venomic workflow to quickly identify its native peptides and proteins. The application of the intact mass profiling (IMP) revealed 80 different molecular masses (Figure 1, Table S1). Proteins could be detected up to a size of ~31 kDa: 9 proteins in a range of 13–15 kDa and 6 in the range of 21–31 kDa. Dominant mass signals of small peptides at *m*/*z* 430–617 were observed, at early gradient retention times, in peaks 1–7. The small molecular determinants could be annotated manually and identified as members of the bradykinin-potentiating peptides-related peptides (BPP-RP) (Figures S1–S9) [41,42]. The peaks 8–15 mainly exhibited masses of 7–8 kDa, putatively identified as disintegrins (DI) by IMP and later confirmed by BU annotation. While the components with molecular masses of ~14 kDa, including peaks 16, 17, 18 and 22, were identified as PLA_2_s, those with molecular masses of 21–28 kDa were suspected as members of the svSP and CTL family (Table S1).

Accordingly, the non-reduced venom of *P. flavoviridis* revealed 9 different proteins belonging to five toxin families: PLA_2_, svMP, DI, BPP-RP and l-amino acid oxidases (LAAO) (Table S1). Interestingly, most sequences were identified as fragments belonging to svMPs, which, however, were attributed to self-digestion, e.g., by metalloproteinases, an effect described previously in vitro for bothropasin and brevilysin H6 [43].

Three different BPP-related peptides were annotated, containing a characteristic pyro-glutamylated N-terminus (Table S1). Additionally, five protein masses were detected as full-length proteins. For each of these, a disintegrin and the four PLA_2_s were annotated, with different modifications (Table S1). Since these mass differences to the expected amino acid sequence were indicated as parts of a longer fragment and did not have a distinct position, we assume the identification of closely related isoforms.

In order to further increase the number of annotations and assignments achieved so far, a chemically reduced venom sample was measured (Figure S10). The reduction of cystines, which are important post-translational modifications (PTMs) in snake venoms, breaks up the tertiary structures and leads to a better fragmentation and *de novo* sequencing [39]. By means of the reduced TD approach, we detected, among other fragments, proteoforms of two 14 kDa toxins. One CTL, with a monoisotopic molecular mass of 14,400.38 Da, was identified as a 31.11 Da lighter variant of the so-called IX/X-BP CTL. The second mass (13,921.36 Da) belongs to the PLA_2_ family and was annotated as a proteoform of the basic phospholipase, A_2_ PL-X (13,971.41 Da). The reductive workup of venom with *tris*(2-carboxyethyl)-phosphine (TCEP) ultimately leads to conformational changes in the proteins and thus to differences in retention time. Therefore, a peak assignment, in comparison to the native TD total ion chromatogram (TIC) nomenclature, was not possible.

Except for the BPP-RP, other isoforms of venom proteins were only detectable as fragments. It is worth mentioning that, of the 73 TD assignments of the reduced venom, only three were annotated as internal fragments, while 54 belong to the N-terminal end, and only 13 belong to the C-terminal end of the compared sequences. The *de novo* sequences were assigned to seven toxin families (Table 1). The high number of observed fragments could be an effect of the aforementioned digestion by metalloproteinases, and thus an accurate peak annotation, in correlation with the native venom TD, was impeded.

### 2.2. Bottom-Up Analysis

**Figure 2:**
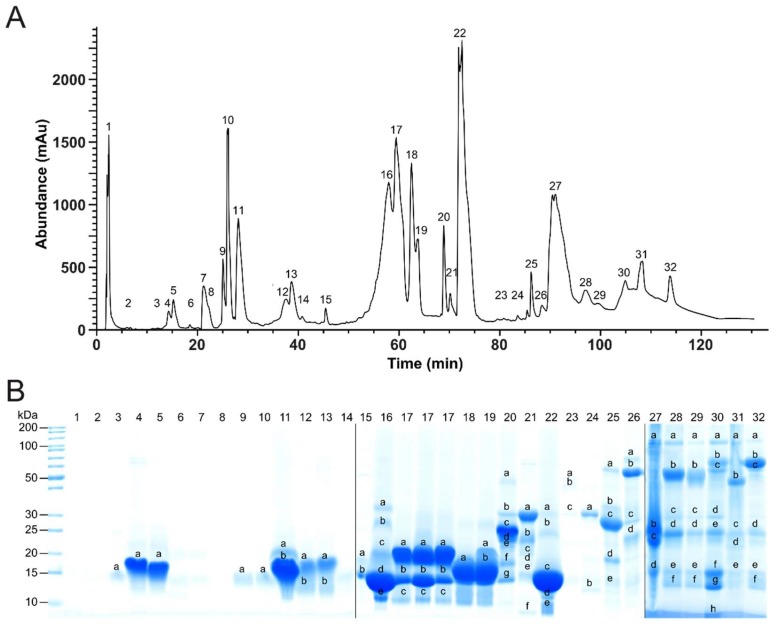
HPLC *P. flavoviridis* venom profile and fractions, separated by SDS-PAGE. (**A**) Crude venom from *P. flavoviridis* was separated on a Supelco Discovery BIO wide Pore C18-3 RP-HPLC column, and components were detected at λ = 214 nm. Collected fractions (assigned above the correlated peaks) were dried, and (**B**) separated by SDS-PAGE under reducing conditions (Coomassie staining). Alphabetically marked bands per line were excised for a subsequent tryptic in-gel digestion.

**Figure 3:**
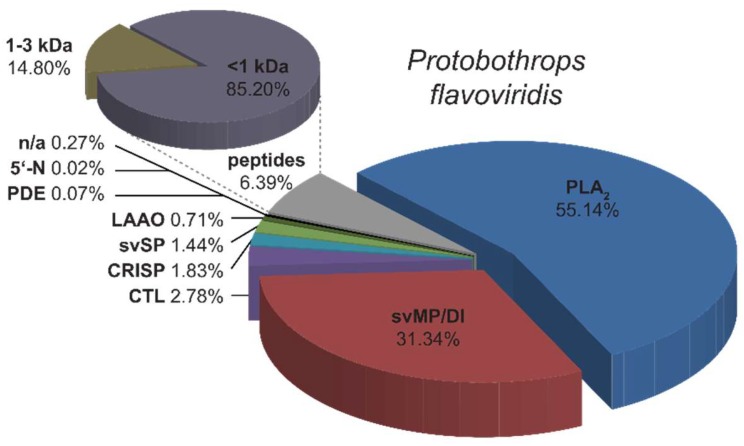
Semi-quantitative venom composition of *P. flavoviridis*. Relative occurrence of different toxin families and peptide content in *P. flavoviridis*: Phospholipases A_2_ (PLA_2_, blue), snake venom metalloproteinases and disintegrins (svMP/DI, red), C-type lectin-like proteins (CTL, violet), cysteine rich secretory proteins (CRISP, light blue), snake venom serine proteases (svSP, light green), l-amino acid oxidases (LAAO, dark green). Phosphodiesterases (PDE), 5′-nucleotidases (5′-N) and some unidentified proteins (n/a) (combined in black and peptides in grey) are less abundant. Groups of different peptide sizes are summarized in an additional pie chart percental related to the total peptide content. They were clustered to <1 kDa (dull purple) and 1–3 kDa (dull brown) parts.

The TD approach gave a first and quick overview of lower molecular mass peptides and proteins, even of less prominent components, as constituents of the venom. A severe limitation of the TD approach, however, is that proteins beyond a molecular mass of ~30 kDa are hardly detectable. This requires complementary analytics by a bottom-up approach: HPLC fractionation (Figure 2A) of the venom is followed by SDS-PAGE (Figure 2B) and a tryptic in-gel digestion of protein bands. The subsequent MS *de novo* sequencing and semi-quantitative analysis led to the identification of the following toxin families: 55.1% phospholipases A_2_ (PLA_2_), 31.3% snake venom metalloproteinases and disintegrins (svMP/DI), 2.8% C-type lectin-like proteins (CTL), 1.8% cysteine-rich secretory proteins (CRISP), 1.4% snake venom serine proteases (svSP), 0.7% l-amino acid oxidases (LAAO), 0.07% phosphodiesterase (PDE) and 0.02% 5′-nucleotidase (5′-N). While 6.4% of the venom was assigned to peptides, 0.3% could not be annotated (n/a) (Figure 3).

According to the above findings, from components of the venom, the phospholipase A_2_ class formed the biggest part and includes the three most abundant proteins in the whole venom: The highest protein content belongs to the acidic PLA_2_ 1 (16.6%), eluting as fraction 22. Secondly, the basic PLA_2_ BP (10.4%) was identified in fraction 16 but could not be clearly assigned as PLA_2_ basic protein I or II (BPI, BPII), because of their high similarity, which differs only in an N58D exchange as a single mutation [44]. The third protein is PLA-N(O) (7.9%) of fraction 17, which is a PLA-N K121N isoform (Uniprot-ID: S6BAM8), previously documented with respect to the *P. flavoviridis* population on Okinawa Island [14].

The svMP and DI members were detected at early retention times (HPLC fractions 4 and 5), but also formed the dominating protein classes at later retention times (fractions 27, 28, 32) (Table S1). Because svMP could include DI domains, both toxin families were combined in the final composition as the svMP/DI part, thus forming the second most abundant group. The annotated sequences from fractions 4–13 belong to svMP P-II disintegrin domains, but with ~15 kDa, the observed molecular masses, determined from the SDS gel, appear too low for svMP P-II (expected molecular masses 30–60 kDa) and too high for single disintegrins (7–8 kDa). It is conceivable that the identified disintegrin domains originate from degraded P-II or P-III metalloproteinases and contain neighboring sequence parts. This could mean that the Habu venom contains truncated or auto-digested versions of svMP P-II or P-III, which which is also known from other svMPs [43]. This autolysis was firstly observed for the two svMPs, HR1A and HR1B, isolated from the *P. flavoviridis* venom [45,46].

The CRISPs represent a minor part of the venom (1.8%) and were only found in fractions 20 and 21. The BU-annotated triflin revealed, in the IMP, a molecular mass of 24,767 Da, with a −16 Da shift to the expected 24,783 Da. The transcriptomic data of *P. flavoviridis* include another CRISP (Uniprot-ID: T2HP25), whose amino acid sequence differs from triflin (Uniprot-ID: Q8JI39) in two mutations: D110N and I113V. This isoform has an average molecular mass of 24,768 Da and would correspond to our observation. To our knowledge, this proteoform, herein termed triflin-II, has not yet been described at the proteomic level.

Another protein, validated by its molecular mass, is the flavoxobin (Uniprot-ID: P05620), which belongs to the low abundant svSP family. This thrombin-like protease is the main part of fraction 25 and, with 0.8%, represents more than half of the svSP venom content, followed by svSP2 (Uniprot-ID: O13057) with 0.3%. In summary, the combination of three mass spectrometric methods, BU, TD and IMP, led to the annotation of specific isoforms (Table 2).

**Figure 4:**
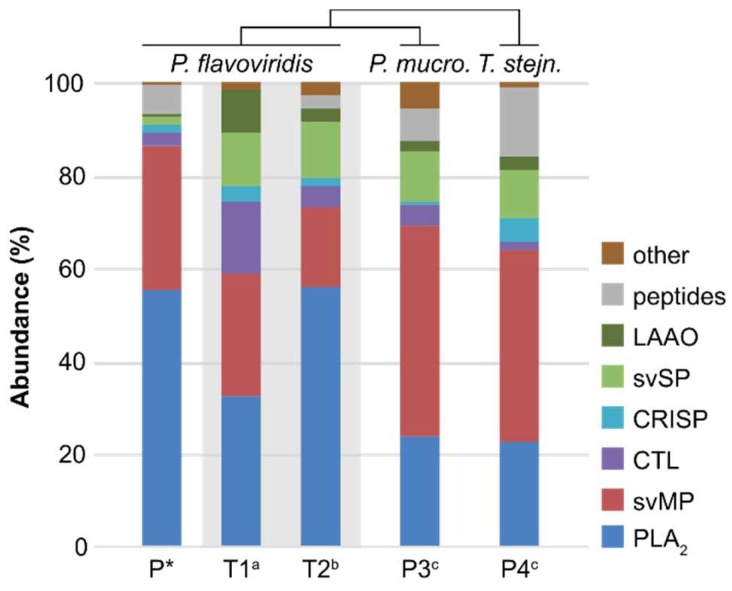
Venomic data of *P. flavoviridis* in comparison to the closed related *P. mucrosquamatus* and *T. stejnegeri*. Venom compositions of three proteomic pit viper analyses (P; quantified at 214 and 215 nm) and two venom gland transcriptomic analyses of *P. flavoviridis* (T1 and T2) in fragments per kilobase million (FPKM %). The asterisked data set P is the result of this study. “Other” represents, in all three *P. flavoviridis* data, phosphodiesterases, 5′-nucleotidases and components that were not annotated. Additionally, in the case of T1 and T2, galactose-binding lectins, nerve growth factors, phospholipases B, glutaminyl cyclases, vascular endothelial growth factor-like proteins, as well as less abundantly detected families, with <0.01%, are included. For *P. mucrosquamatus* (P3, *P. mucro.*), “other” represents trimucrotoxin, and, for *T. stejnegeri* (P4, *T. stejn.*), “other” represents snake venom vascular endothelial growth factors. The origins of toxin ratios are marked alphabetically: a [37], b [31], c and d [50]. The phylogentic relationship is based on [15,51].

In contrast to the proteomic data presented herein, the Habu is already known to have two transcriptomic compositions from an mRNA analysis of the venom glands [31,37]. They exhibit small analogies between the protein abundances and the fragments per kilobase million (FPKM %) of the main toxin families, but also remarkable differences regarding the proportional distribution (Figure 4). Both transcription analyses (T1, T2 in Figure 4) show PLA_2_ as the highest expressed gene family, followed by svMP, which coincides with our proteomic data (P* in Figure 4). On the other hand, in T1, the PLA_2_s form ~30%, while the amounts of P and T2 are comparable (~55%). With 17.3%, the svMP gene expressions of T2 are lower than T1 and the protein amounts, identified in the proteome (~30%). Interestingly, the proteomic and transcriptomic compositions are correlated in less represented groups: CRISP (2–4%) and PDE (0.2–0.1%). In the other three families (CTL, svSP, LAAO), our measured proteomic levels are much lower than the mRNA level. The data for CTL show an expression up to five-fold and, for svSP, eight-fold, higher than that of the protein levels found. There is a considerable difference between the observed 0.7% of LAAOs in our study and T1 as well as T2, which show a four to 13-fold larger amount, accounting for up to 9.1% of the complete transcriptome. Due to the fact that modern analysis can detect small amounts of RNA, very low concentrated targets can usually be observed, but are not necessarily visible in proteomic approaches. All three studies identified PDEs and 5′-nucleotidases, with abundances of >0.2%. In the case of T1 and T2, further families, in a range of 0.65–0.01%, were observed, but not in the venomic proteome P: Galactose-binding lectins, nerve growth factors, phospholipases B, glutaminyl cyclases, vascular endothelial growth factor-like proteins, as well as less abundantly detected families, with <0.01% of the total transcript abundance [31,37]. This underlines that reflecting the individual proteomic and transcriptomic compositions could not easily be compared with independent venom analyses of a species. This has also been shown in studies on other members of *Viperidae* and *Elapidae* [47,48,49]. However, these two approaches in combination mutually represent a powerful aid for protein annotation and identification.

The venom of two related pit vipers show analogies in their composition to *P. flavoviridis*. This concerns *Protobothrops* (or *Trimeresurus*) *mucrosquamatus* (*P. mucrosquamatus*), named the Taiwan Habu, as well as the *Trimeresurus stejnegeri* (*T. stejnegeri*) (indicated with P3, P4 in Figure 4) [15,51]. As for *P. flavoviridis*, they can be found on island habitats, e.g., Taiwan, in the south-west of Okinawa at the end of the Japanese island chain, and the *P. mucrosquamatus* was also observed directly on Okinawa [15,51,52]. Like the Habu from Japan, these two vipers belong to the medically important venomous snakes in their Taiwanese habitat and are responsible for significant envenomations and deaths over the last decades [53,54].

For all three snakes, the main venom families are svMPs and PLA_2_s, followed by CTLs, CRISPs, svSPs, and LAAOs with abundances of <15%. While of the above-mentioned *P. flavoviridis* venom (P* in Figure 4) comprises a big portion, i.e., >55% PLA_2_s and >30% svMPs, the venoms of P3 and P4 are alike to each otther. The main protein share of both venoms (P3 and P4 in Figure 4) is contributed by svMP, with >40%, while the PLA_2_s (~25%) only constitute the second most abundant protein component and thus stand in contrast to P*. The lesser protein families are twofold more abundant in P3 and P4, and reflect a broader diversity of the venomous components in theses snakes. Particularly, the svSP with >10% seemed to be more abundant in the *P. mucrosquamatus* and *T. stejnegeri*, than in the *P. flavoviridis*.

### 2.3. Bradykinin-Potentiating Peptides and Snake Venom Metalloproteinase Inhibitors

Besides the previously mentioned protein families, various bradykinin-potentiating peptides (BPP) and snake venom metalloproteinases inhibitors (svMP-i) are further constituents of snake venoms. The strong vasoactive effect of bradykinin, a substrate of the angiotensin-converting enzyme, was discovered in the late 1940’s in studying the *Bothrops jararaca* venom, and this discovery indicates that research on snake venoms can lead to impressive developments in the drug development field [55,56]. The identification of a small peptide in the same venom, which increased the effects of Kinin, was the first BPP [57]. This facilitated the design of hypertension drugs based on the structure of a snake toxin structure [58]. Today, a multitude of different snake BPPs are known, and, with the progress in the field of venomics, this number is still increasing.

By means of TD and IMP analytics, in total, we identified 5 different BPP-RP bearing an N-terminal pyro-glutamate (pE): pEQWMPGGRPPHHIPP (Figure S1) and pESKPGRSPPISP (Figure S2). Until now, the presence of both peptides was only hypothesized by transcriptomic data on *P. flavoviridis* [37,59].

In the peak of the BPP, pESKPGRSPPISP (peak 5, Figure S1), by means of MS/MS, two C-terminally truncated versions were identified: The 11mer peptide, pESKPGRSPPIS (Figure S3), and the 10mer, pESKPGRSPPI (Figure S4). Comparable to the peptide, pEQWMPGGRPPHHIPP, the pEQWSQGRPR peptide (Figure S5) of peak 2 shows a trimeric N-terminal sequence (pEQW), which, as a tripeptide, is known for its inhibition of svMP.

To minimize the risk of a self-degradation by metalloproteinases, also present in high concentrations in the herein studied venom, snakes secrete small trimeric peptides. These svMP inhibitors are processed from the same precursor, like the BPPs, and also contain an N-terminal pyroglutamate [60]. In summary, we could identify three different svMP-is that represent the main components of the TIC: Peak 4 (pEKW, *m*/*z* 444.22), peak 6 (pENW, *m*/*z* 430.17) and peak 7 (pEQW, *m*/*z* 444.18) (Figures S6–S8). Another prominent molecular mass signal beneath the main peak, *m*/*z* 444.18, was detected at *m*/*z* 427.13 and could be identified as pEEW, which is the deaminated form of peak 7 pEQW (Figure S9). Previously, these three svMP-is were further isolated from the closely related Taiwanese Habu and revealed their strong inhibitory activity [61].

### 2.4. Cytotoxicity Test

It is well-known that the snake venom of *P. flavoviridis* causes strong cytotoxic effects and various isolated toxins, e.g., LAAO Okinawa Habu apoxin protein-1 (OHAP-1) in glioma cells [62,63], exhibiting in vivo apoptotic activities. The Habu venom was monitored in this study against several human cancer cell lines and, therefore, the cytotoxicity was determined for cancerous (SH-SY5Y, MDA-MB-231, A549, PANC1, HeLa, PC-3) as well as non-cancerous (HEK293) cells by the MTT assay. The IC_50_ values range from ~1 to >50 μg/mL (Table 3).

The highest inhibition of proliferation was found against HEK293 (1.02 μg/mL) and SH-SY5Y (4.7 μg/mL) cells (Figure S11). SH-SY5Y, as the most sensitive cancer cell line, has been selected for further screenings with single RP-HPLC venom fractions. Regarding the most abundant molecular mass in a collected fraction, we indicated the IC_50_ in µg/mL and µM. While various tested fractions were found to be active, fractions 4 and 7/8 were the most potent, with IC_50_ values of 0.7 and 0.9 μg/mL (Table 4). The main compound of the fraction 4 is the svMP-i pEKW, while the pEQW containing fraction 7/8 is similarly potent. According to these results, the *P. flavoviridis* venom, svMP-is, exhibits remarkable effects on SH-SY5Y cells, while, until now, only the correlating svMP of most *Protobothrops* venoms were known to have an important role in envenomation-related pathologies [46,64]. Regarding the identified families, PLA_2s_, as the main venom part, are most active in fraction 15 (PLA_2_, 2.2 µg/mL), in combination with a CTF-II-like disintegrin, against neuroblastoma cells. An induced caspase-independent apoptosis by another PLA_2_ (BP-II) in a leukemia cell was previously shown [63]. The PLA_2_ fractions (16, 17, 18, and 19) as well as the CRISP triflin-II fraction 20 revealed a moderate growth inhibition (16 to 30 µg/mL) with respect to the mass concentration.

**Figure 5:**
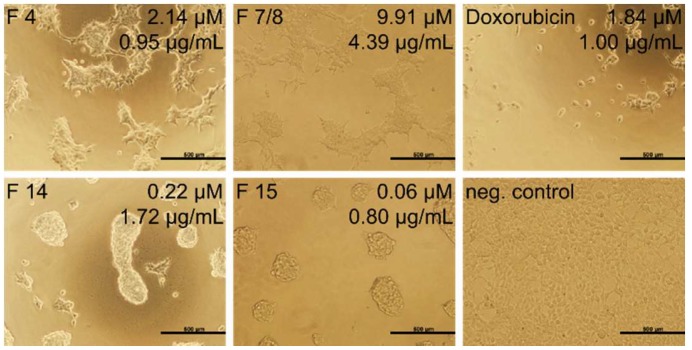
The most active *P. flavoviridis* venom fraction treatment of SH-SY5Y cells. Single RP-HPLC venom fractions of *P. flavoviridis*, with the aforementioned mass concentration in µg/mL and molar concentration in µM, were tested against human neuroblastoma SH-SY5Y cells. Images were taken after 48 h of treatment at 37 °C. Doxorubicin was used as a positive cytotoxic control drug and no stimulation as a negative control.

Fractions 14 and 15 show an IC_50_ of 0.16 and 0.53 µM, respectively, and both are more active than the positive control, doxorubicin (1.53 µM). Likewise, triflin-II (25 kDa), with an IC_50_ of 0.63 µM, is highly potent. The effect on SH-SY5Y cells of the most active fractions, 14 and 15, as well as peptide fractions, 4 and 7/8, is shown by microscopic imaging (Figure 5). Further tested fractions, with different concentrations, are overviewed (Figure S12). Fractions 7/8 and 11–15 indicate, against SH-SY5Y cells at higher concentrations, a disintegrine-like conglomeration effect (Figure S12), which correlates with the observation of svMP/DI in these fractions by BU, IMP and the TD, like the disintegrin CTF-II proteoform (Table S1). Future studies will focus on the mechanism of the *P. flavoviridis* venom action in SH-SY5Y cells, notably due to DI and svMP-i.

## 3. Discussion

In this contribution, we report on the first quantitative mass spectrometry-guided proteomic snake venom analysis of the pit viper, *P. flavoviridis*, one of the most feared and life-threatening snakes in Japan. The combination of all three mass spectrometric methods (BU, TD and IMP) facilitates the annotation of isoforms, respectively based on databases even without the full sequencing of every protein. The combined data reveal that PLA_2_s (55.1%) comprise the major part of the venom, with PLA_2_ 1, PLA_2_ BP I/II and PLA-N(O) as main representatives, as well as the svMP/DI group and several minor represented toxin families, like the svSP with flavoxobin and svSP2. For the first time, a CRISP triflin-homolog, named triflin-II, was observed at the proteomics level. The top-down approach to identify proteins as well as peptides was reliable for toxins with molecular masses up to ~30 kDa. Venom compounds, in a range of *m*/*z* 338.1 to 30,384.7, were detected by the intact mass profiling, with the same restrictions for high masses as in the top-down approach, while the SDS-PAGE exhibits proteins over 100 kDa.

An analytical correlation of the proteomes, with two further pit viper venoms, shows that a close relationship and similar habitats are not predictive of the venom compositions of related species. This emphasizes the importance of an individual snake venom analysis to reveal the specific components, e.g., those found for the various PLA_2_s of *P. flavoviridis*. Furthermore, these finding may help the development of targeted snake bite therapies.

Interestingly, venom-peptides display various bioactivities, and their antiproliferative and cytotoxic properties may also contribute to therapies in the treatment of cancer. This is corroborated by the fact that several venom-based drugs are medically assessed, e.g., chlorotoxin, against different tumor cell types and the integrin αvβ3-targeted cancer therapy [65,66,67]. In this study, we screened fractions of the *P. flavoviridis* crude venom for cytotoxic effects against cancer cells. Accordingly, cytotoxicity assays of crude venom and growth inhibition by venom fractions revealed promising results against SH-SY5Y neuroblastoma cells. The observed effect of svMP-i tripeptide, containing fractions on cell growth inhibition, appears very interesting from a pharmacological viewpoint as well as for a future mode of action studies, and it reveals a new facet of the bioactivity of these small venom components. Moreover, we identified the three highly potent protein fractions, 14, 15 and 20, with IC_50_ values, ranging between 0.16 and 0.63 µM, which are 2–10× fold lower than the cytostatic doxorubicin. These aspects, concerning the bioactivity of components of the *P. flavoviridis* venom on neuroblastoma cells, might be helpful in the development of future anti-cancer treatments.

Through the wide range quantification of the Okinawa habu venom, we could extend the picture of the Japanese venomous snakes, of which only a few detailed analyses have addressed the whole venomic level.

## 4. Materials and Methods

### 4.1. Sample Preparation and System Setup

The pooled Habu venom of six *P. flavoviridis* specimens (four females, two males) was purchased from the Kentucky Reptile Zoo (Slayde, KY, USA) and kindly provided by Professor Dr. Johannes A. Eble (University of Münster, Germany). The crude venom (with a final concentration of 10 mg/mL) was dissolved in 10 µL HFo (1% (*v*/*v*) and centrifuged at 20,000× *g* for 5 min. Then, 30 µL of citrate buffer (0.1 M, pH 4.3) was added. One half of the sample (20 µL) was chemically reduced by adding 10 µL of 0.5 M *tris*(2-carboxyethyl)-phosphine (TCEP) and incubated for 30 min at 65 °C, while 10 µL ultra-pure water was added to the other half, as a non-reduced/native sample. All samples were centrifuged at 20,000× *g* for 5 min and submitted to IMP (native) and TD venomics (native, reduced): HPLC-high-resolution (HR) ESI-MS/MS measurements were performed on a LTQ Orbitrap XL mass spectrometer (Thermo, Bremen, Germany) coupled to an Agilent 1260 HPLC system (Agilent, Waldbronn, Germany) using a Supelco Discovery 300 Å C18 (2 × 150 mm, 3 µm particle size) column. The elution was performed by a gradient of ultra-pure water, with 0.1% formic acid (HFo) (*v*/*v*; buffer A), and acetonitrile (ACN), with 0.1% HFo (*v*/*v*; buffer B), at a flow rate of 1 mL/min. An isocratic equilibration (5% B) for 5 min was followed by a linear gradient of 5–40% B for 95 min, 40–70% B for 20 min, 70% B for 10 min and a re-equilibration with 5% B for 10 min.

ESI settings were: 11 L/min sheath gas, 35 L/min auxiliary gas, spray voltage 4.8 kV, capillary voltage 63 V, tube lens voltage 135 V, and capillary temperature 330 °C. The data-dependent acquisition (DDA) mode was used for MS/MS experiments, with 1 μ scans and 1000 ms maximal fill time. The precursor ions were selected, with a range of ±2 *m*/*z* and after two repeats within 10 s, excluded with ±3 *m*/*z* for a duration of 20 s. Three scan events were performed, with a normalized collision-induced dissociation (CID) energy of 30% and 35%, and a higher-energy collisional dissociation (HCD) with 35% collision energy.

### 4.2. Intact Mass Profiling (IMP)

For IMP, the mass spectrometric data were inspected via the Xcalibur Qual Browser (Thermo Xcalibur 2.2 SP1.48, Waltham, MA, USA), and the deconvolution of isotopically resolved spectra was carried out using the XTRACT algorithm of Xcalibur Qual Browser. The protein assignment was done by comparison with the retention times obtained from the HPLC runs. Sequence annotations and molecular mass comparisons with protein database entries of *P. flavoviridis* (taxid: 88087) were performed manually.

### 4.3. Top-Down (TD) Venomics

The top-down analytical data were obtained based on the protocol of Petras et al., 2016 [68], with the following alterations: Data were inspected with the Qual Browser (Thermo Xcalibur 2.2 SP1.48) and prepared based on the TopPIC workflow. The .raw data were converted to a centroided .mzXML using the MSconvert of the ProteoWizard package (http://proteowizard.sourceforge.net), version 3.0.10577. The .mzXML data were deconvoluted to an .msalign file using MS-Deconv (http://bix.ucsd.edu/projects/msdeconv), version 0.8.0.7370 (maximum charge 30, maximum mass 50,000, *m*/*z* tolerance 0.02, s/n ratio 1.0). The final sequence annotation was performed by TopPIC (http://proteomics.informatics.iupui.edu/software/toppic/), version 1.0.0, with a targeted search type, error tolerance of 15 ppm and an E-value cutoff at 0.01 by E-value computation.

Variable modifications were: Acetylation of Lys (+42.010565 Da), phosphorylation of Ser, Thr and Tyr (+79.966331 Da), oxidation of Met (+15.994915 Da), N-terminal methylation (+14.015650 Da), N-terminal pyro-Glu formation of Glu (−18.010565 Da) and Gln (17.026549 Da), *N*-acetylhexoseamine formation of Arg (HexNAc +203.079373 Da) and, in the case of non-reduced samples, a dehydrogenation of Cys (−1.007825 Da for each Cys), rendering Cys-Cys bridges. A maximum of two unexpected modifications was allowed, with a 500 Da maximal mass.

The sequences were matched against a protein NCBI database of *P. flavoviridis* (taxid: 88087, 852 entries, 8 September 2017), manually validated and graphically visualized using the MS and MS/MS spectra of the Xcalibur Qual Browser.

### 4.4. Bottom-Up (BU) Venomics

Lyophilized crude venom (4 mg) was dissolved, with a final concentration of 20 mg/mL, in aqueous 3% (*v*/*v*) and ACN, with 1% (*v*/*v*) HFo. The solution was centrifuged at 20,000× *g* for 5 min, and the supernatant was loaded onto a semi-preparative reversed-phase HPLC with a Supelco Discovery BIO wide Pore C18-3 column (4.6 × 150 mm, 3 µm particle size) using an Agilent 1260 Low Pressure Gradient System (Agilent, Waldbronn, Germany). As a gradient buffer, ultra-pure water, with 0.1% (*v*/*v*) HFo (buffer A), and ACN, with 0.1% (*v*/*v*) HFo (buffer B), were used, with a flow rate of 1 mL/min. An isocratic equilibration (5% B) for 5 min was followed by toxin elution, with a linear gradient of 5–40% B for 95 min, 40–70% B for 20 min and 70% B for 10 min.

Absorbance was measured at λ = 214 nm using a DAD detector, and 1 mL fractions were automatically collected. Peaks of the chromatograms were manually pooled and vacuum dried (Thermo Speedvac, Bremen, Germany). Fractions were submitted to SDS-PAGE under reducing conditions [69]. Coomassie (Blue G250, Serva, Heidelberg, Germany) stained protein bands were cut, in-gel reduced with fresh dithiothreitol (100 mM DTT in 100 mM ammonium hydrogencarbonate, pH 8.3, for 30 min at 56 °C) and alkylated with fresh iodoacetamide (55 mM IAC in 100 mM ammonium hydrogencarbonate, pH 8.3, for 20 min at 25 °C). An in-gel trypsin (Thermo, Rockfeld, IL, USA) digestion was performed (6.7 ng/µL in 10 mM ammonium hydrogencarbonate with 10% (*v*/*v*) ACN, pH 8.3, for 18 h at 37 °C, with 0.27 µg/band). The peptides were extracted with 100 µL aqueous 30% (*v*/*v*) ACN, with 5% (*v*/*v*) HFo, for 15 min at 37 °C, and the supernatants were vacuum dried, re-dissolved in 20 µL aqueous 3% (*v*/*v*) ACN, with 1% (*v*/*v*) HFo, and submitted to LC-MS/MS analysis.

The analytics of tryptic peptides were performed using a reversed-phase Grace Vydac 218MSC18 column (2.1 × 150 mm, 5 µm particle size) under the control of an Agilent 1260 HPLC system (Agilent Technologies, Waldbronn, Germany). The HPLC separation operated with a flow rate of 0.3 mL/min. After an isocratic equilibration (5% B) for 1 min, the peptides were eluted, with a linear gradient of 5–40% B for 10 min and 40–99% B for 3 min, washed with 99% B for 3 min and re-equilibrated in 5% B for 3 min.

MS experiments were performed on an Orbitrap XL mass spectrometer (Thermo, Bremen, Germany), with R = 15,000 at *m*/*z* 400 and at a maximum filling time of 200 ms for the first product ion scans. MS/MS fragmentation of the most intense ion was performed in the LTQ using a collision-induced dissociation (30 ms activation time); the collision energy was set to 30% and 35%. The precursor ions were selected, with a range of ±2 *m*/*z*, and, after two repeats within 10 s, excluded with ±3 *m*/*z* for a duration of 20 s.

LC-MS/MS data files (.raw) were converted into mgf files via MSConvert GUI of the ProteoWizard package (http://proteowizard.sourceforge.net; version 3.0.10577) and annotated by DeNovo GUI 1.15.11 [70], with carbamidomethylated cysteine (+57.021464 Da) as a fixed modification. Variable modifications were the acetylation of lysine (+42.010565 Da) and phosphorylation of serine and threonine (+79.966331 Da).

The peptide sequences were matched against a non-redundant protein in the NCBI database of *P. flavoviridis* (taxid: 88087) using BLASTP (http://blast.ncbi.nlm.nih.gov).

### 4.5. Relative Toxin Quantification

The percentage composition of the venom ingredients was calculated on the basis of a combination of the RP-HPLC chromatogram and SDS-PAGE evaluation [71,72]. The peak integrals at UV_214nm_ were measured in comparison to the total sum of peak integrals. In the case of multiple component elution in an HPLC fraction identified by SDS-PAGE staining, the integrated density ratio of the stained bands was respectively used for the emphasis of peak integrals.

### 4.6. Data Accessibility

Mass spectrometry proteomics data (.mgf, .raw and output files) have been deposited at the ProteomeXchange Consortium [73] (http://proteomecentral.proteomexchange.org) via the MassIVE partner repository, under the project name “Venomics of the Okinawa Habu pit viper, *Protobothrops flavoviridis*,” and the data set identifier, PXD009414.

### 4.7. Cell Culture and In Vitro Cytotoxicity Assay

Human cells were purchased from ATCC (Manassas, VA, USA) and cultivated in Dulbecco’s modified Eagle’s medium, F-12 (DMEM/F-12, 10% fetal bovine serum (FBS), 2 mM/L glutamine, 100 U/mL penicillin, 100 mg/mL streptomycin) (Gibco, Carlsbad, CA, USA). The following cell lines were used: HEK-293 (human embryonic kidney), SH-SY5Y (neuroblastoma), MDA-MB-231 (breast epithelial adenocarcinoma), A549 (human alveolar adenocarcinoma), PANC1 (pancreas adenocarcinoma), HeLa (human cervix adenocarcinoma) and PC-3 (human prostate adenocarcinoma). In vitro cytotoxicity assays were performed with crude venom and HPLC fractions using a modified 3-(4,5-dimethyl-2-thiazolyl)-2,5-diphenyl-2H-tetrazoliumbromide) (MTT). The protein concentrations in saline were estimated using a BCA protein assay kit (Thermo-Scientific, Darmstadt, Germany) with a 595 nm ultraviolet (UV)-visible spectrophotometer (VersaMax, Molecular Devices, San Jose, CA, USA). Bovine serum albumin was used as a standard for the calibration line.

A modified MTT assay was used to determine the cytotoxicity of the snake venom. Therefore, 1 × 10^5^ cells/mL were seeded in a 96-well microtiter plate. After 24 h of cultivation, the cells were treated for 48 h at 37 °C with crude venom, with venom fractions or doxorubicin as positive cytotoxic control drugs.

The optical density (OD) was measured in triplicates at λ = 570 nm (with a reference wavelength of λ = 690 nm) by UV/Vis spectrophotometry (Thermo, Bremen, Germany). The cell viability was determined with an absorbance of *A*:

(1) $$\mathrm{Viable}\;\mathrm{cells}=\frac{A_{\mathrm{treated}}-A_{\mathrm{blank}}}{A_{\mathrm{untreated}}-A_{\mathrm{blank}}}\cdot 100$$

### 4.8. Morphological Studies

The morphological changes of the cells following treatment with crude venom or single RP-HPLC venom fractions of *P. flavoviridis* were observed under an inverted microscope (Olympus, Toyo, Japan) and compared to the control group following a 48 h treatment.

### 4.9. Half Maximal Inhibition of Growth (IC_50_) Determination

The half maximal inhibition of growth (IC_50_) was calculated based on a sigmoidal curve fitting using a four-parameter logistic model, as compared to that of untreated controls, which was calculated using Prism 5 software (GraphPad5, San Diego, CA, USA). Values are presented at a 95% confidence interval and as the average of three independent measurements.

## Figures and Tables

**Table 1:** Identified proteoforms and fragments of *P. flavoviridis* venom by reduced top-down analytics. Assignment of reduced crude venom components by a top-down (TD) analysis. Sequence tags were obtained *de novo* from MS/MS spectra and identified against a *P. flavoviridis* National Center for Biotechnology Information (NCBI) protein database (taxid: 88087) by TopPIC.

Toxin Families	Protein ID	Highest E-Value	NCBI Accession No.	No. of Sequence Proteoforms
PLA_2_	basic phospholipase A_2_ BP-III	8.21 × 10^−16^	C7G1G6.1	20
	basic phospholipase A_2_ PL-B	7.42 × 10^−15^	P59265.1	11
	phospholipase A_2_	7.85 × 10^−10^	1202299A	11
	basic phospholipase A_2_ PL-X	8.35 × 10^−10^	P06860.1	5
	basic phospholipase A_2_ PLA-A	2.98 × 10^−14^	P59264.1	3
	basic phospholipase A_2_ BP-II	3.70 × 10^−6^	P0DJJ9.1	2
	basic phospholipase A_2_ BP-I	8.90 × 10^−6^	P0DJJ8.1	2
	phospholipase A_2_	4.17 × 10^−3^	BAA01561.1	2
	basic phospholipase A_2_ PL-Y	2.37 × 10^−8^	Q90Y77.1	1
svMP	zinc metalloproteinase/disintegrin	1.29 × 10^−6^	P18619.2	3
	P-II metalloprotease	5.12 × 10^−5^	BAN89360.1	1
	snake venom metalloproteinase HR2a	2.16 × 10^−3^	P14530.3	1
	snake venom metalloproteinase trimerelysin-II	3.12 × 10^−3^	P20165.3	1
DI	disintegrin CTF-II	4.91 × 10^−7^	P23323.1	3
	cytotoxic factor	9.96 × 10^−8^	AAB19943.1	1
	disintegrin triflavin	4.67 × 10^−4^	P21859.1	1
LAAO	l-amino acid oxidase	1.21 × 10^−6^	BAP39950.1	1
	l-amino acid oxidase	1.28 × 10^−4^	BAN82013.1	1
CTL	coagulation factor IX/X-binding protein	1.21 × 10^−6^	1IXX_F	1
BPP related	Bradykinin-potentiating and C-type natriuretic peptides	4.93 × 10^−6^	BAP39952.1	1
CRISP	CRISP Family Ca-Channel Blocker	7.52 × 10^−5^	1WVR_A	1

**Table 2:** Identification of several venom isoforms by mass comparison. The bottom-up method (BU) in combination with the intact mass profiling (IMP) leads to a database-oriented identification of the isoforms in the crude venom. The theoretical mass includes oxidized cysteines for predicted Cys-Cys bridges.

Fraction	Toxin Family	Isoform Identity by		UniProt-ID for IMP	Average Mass in Da	
		BU	IMP		Observed	Theoretical
16	PLA_2_	BPII	BPII	P0DJJ9	13,752.4	13,752.1
			BPI	P0DJJ8		13,753.1
17	PLA_2_	PLA *	PLA-N(O)	S6BAM8	14,020.2	14,019.2
18	PLA_2_	PLA *	PL-Y	Q90Y77	13,944.3	13,945.2
20	CRISP	triflin	triflin-II	T2HP25	24,767.2	24,767.9
22	PLA_2_	PLA_2_ 1	PLA_2_ 1	P06859	13,764.0	13,764.6
25	svSP	flavoxobin	flavoxobin	P05620	25,686.4	25,687.4

* general identification as a member of the family.

**Table 3:** IC_50_ values of *P. flavoviridis* venom against human cell lines. The half maximal inhibitory concentrations (IC_50_ in µg/mL) were determined for the crude venom of *P. flavoviridis* against one non-cancerous (HEK-293) and six cancerous human cell lines. Error mean in ±SD.

Cell Line	*P. flavoviridis* IC_50_ Values in µg/mL	Doxorubicin IC_50_ Values in µg/mL
HEK-293	1.02 ± 0.02	0.002 ± 0.001
SH-SY5Y	4.68 ± 0.92	0.06 ± 0.02
MDA-MB-231	22.84 ± 2.51	4.98 ± 0.28
A549	38.51 ± 0.13	1.03 ± 0.37
PANC1	>50	0.05 ± 0.01
HeLa	24.78 ± 0.77	1.03 ± 0.25
PC-3	51.55 ± 2.79	2.02 ± 0.46

**Table 4:** IC_50_ values of *P. flavoviridis* venom fractions against SH-SY5Y cells. Single RP-HPLC venom fractions of *P. flavoviridis* were tested against human neuroblastoma SH-SY5Y cells, the half maximal inhibitory concentrations (IC_50_) were determined in µg/mL, and the molar concentration in µM, regarding the most abundant molecular mass, were determined in Da. Doxorubicin was used as a reference and error mean in ±SD.

HPLC Fraction	Molecular Mass in Da	IC_50_	
		in µg/mL	in µM
4	443	0.680 ± 0.002	1.53 ± 0.01
5	1233	4.60 ± 0.24	3.73 ± 0.19
6	429	9.19 ± 1.22	21.42 ± 2.84
7/8	443	0.87 ± 0.05	1.96 ± 0.11
10	7508	49.63 ± 2.46	6.61 ± 0.33
11	7956	45.46 ± 7.45	5.71 ± 0.94
13	7956	17.94 ± 2.96	2.25 ± 0.37
14	7621	4.02 ± 0.24	0.53 ± 0.03
15	14,000 *	2.18 ± 0.12	0.16 ± 0.01
16	13,752	30.57 ± 1.50	2.22 ± 0.11
17	14,020	23.96 ± 2.55	1.71 ± 0.18
18	13,944	38.82 ± 2.82	2.78 ± 0.20
19	14,000 *	17.25 ± 0.98	1.23 ± 0.07
20	24,767	15.57 ± 1.10	0.63 ± 0.04
Doxorubicin	544	0.83 ± 0.20	1.53 ± 0.37

* based on SDS-PAGE size estimation.

## Supplementary Materials

The following are available online. Figures S1–S5: Annotated MS2 spectra of BPP-RP; Figures S6–S9: Annotated MS2 spectra of tripeptidic svMP-I; Figure S10: *P. flavoviridis*-reduced venom, MS TIC, for IMP and TD; Figure S11: *P. flavoviridis* venom, tested for cytotoxicity against different human cell lines; Figure S12: SH-SY5Y cells after 48 h treatment with different *P. flavoviridis* venom fractions.
