# Supplementary material for: Comprehensive Snake Venomics of the Okinawa Habu Pit Viper, Protobothrops flavoviridis, by Complementary Mass Spectrometry-Guided Approaches
Source: Molecules. 2018 Jul 29;23(8):1893. doi: 10.3390/molecules23081893 (PMC6222445; doi:10.3390/molecules23081893)
Supplement: Supplementary file 1 [file molecules-23-01893-s001.zip › molecules-332566-supplementary-figures.pdf]

molecules

MDPI

Article

Comprehensive Snake Venomics of the Okinawa Habu Pit Viper, *Protobothrops flavoviridis*, by Complementary Mass Spectrometry-Guided Approaches

Maik Damm [1,†], Benjamin-Florian Hempel [1,†], Ayse Nalbantsoy [2] and Roderich D. Süssmuth [1,*]

[1] Department of Chemistry, Technische Universität Berlin, 10623 Berlin, Germany; maik.damm@tu-berlin.de (M.D.); benjamin.hempel@chem.tu-berlin.de (B.-F.H.)
[2] Department of Bioengineering, Ege University, 35100 Izmir, Turkey; analbantsoy@gmail.com
* Correspondence: suessmuth@chem.tu-berlin.de; Tel.: +49-30-314-24205

† These authors contributed equal to this work

Supplementary Materials: The following are available online, Figure S1-S5: Annotated MS2 spectra of BPP-RP, Figure S6-S9: Annotated MS2 spectra of tripeptidic svMP-i, Figure S101: *P. flavoviridis* reduced venom MS TIC for IMP and TD, Figure S11: *P. flavoviridis* venom tested for cytotoxicity against different human cell lines, Figure S12: SH-SY5Y cells after 48 h treatment with different *P. flavoviridis* venom fractions.

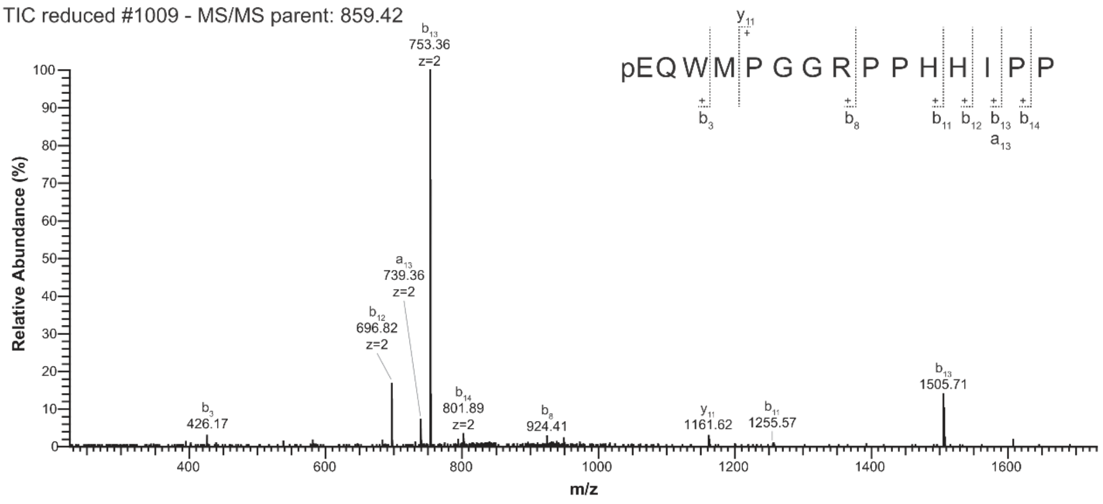

Figure S1. MS2 spectra of pEQWMPGGRPPHHIPP. Representative MS/MS spectra of the *m/z* 859.42 (z=2) precursor ion for the top-down annotation of a BPP.

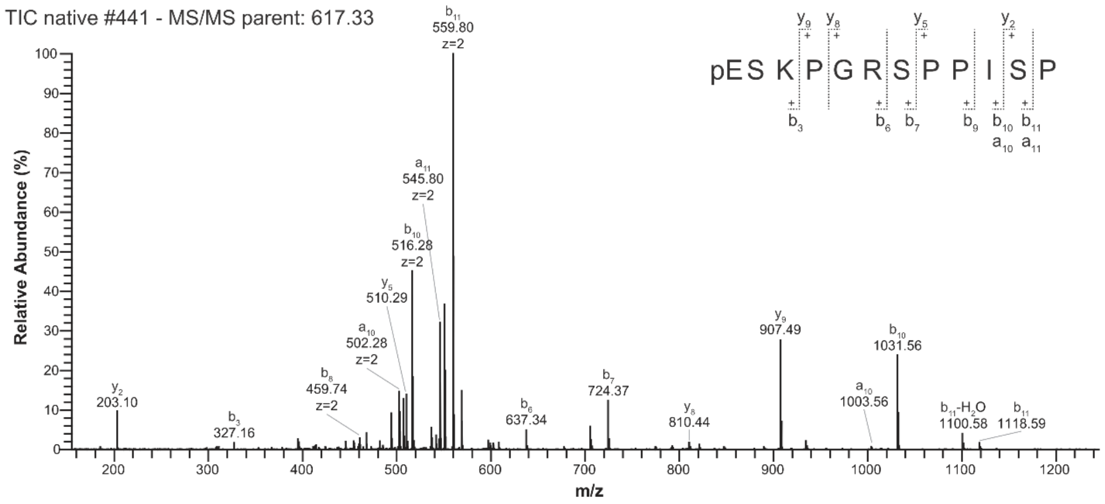

Figure S2. MS2 spectra of pESKPGRSPPISP. Representative MS/MS spectra of the *m*/*z* 617.33 (z=2) precursor ion for the top-down annotation of a BPP.

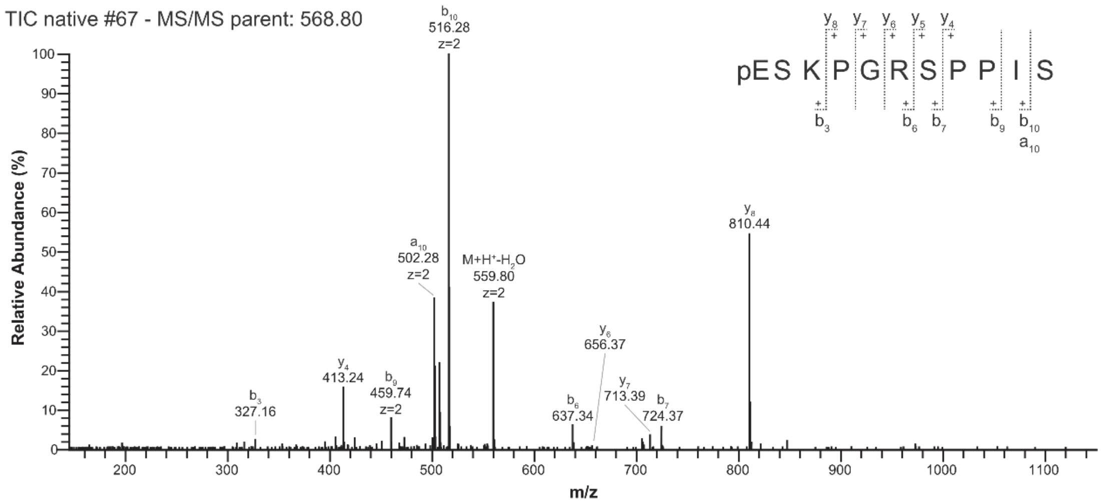

Figure S3. MS2 spectra of pESKPGRSPPIS. Representative MS/MS spectra of the *m*/*z* 568.80 (z=2) precursor ion for the top-down annotation of a BPP.

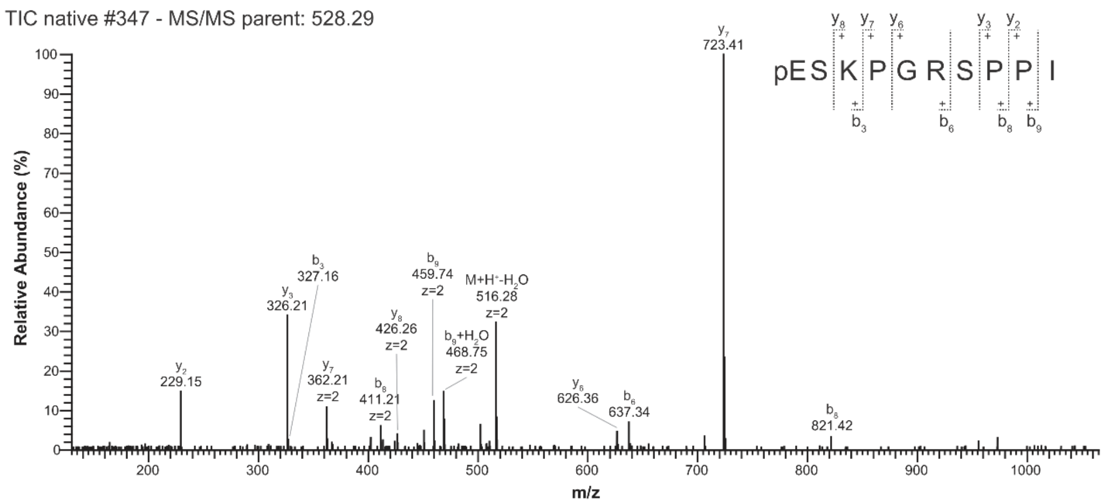

Figure S4. MS2 spectra of pESKPGRSPPI. Representative MS/MS spectra of the *m*/*z* 528.29 (z=2) precursor ion for the top-down annotation of a BPP.

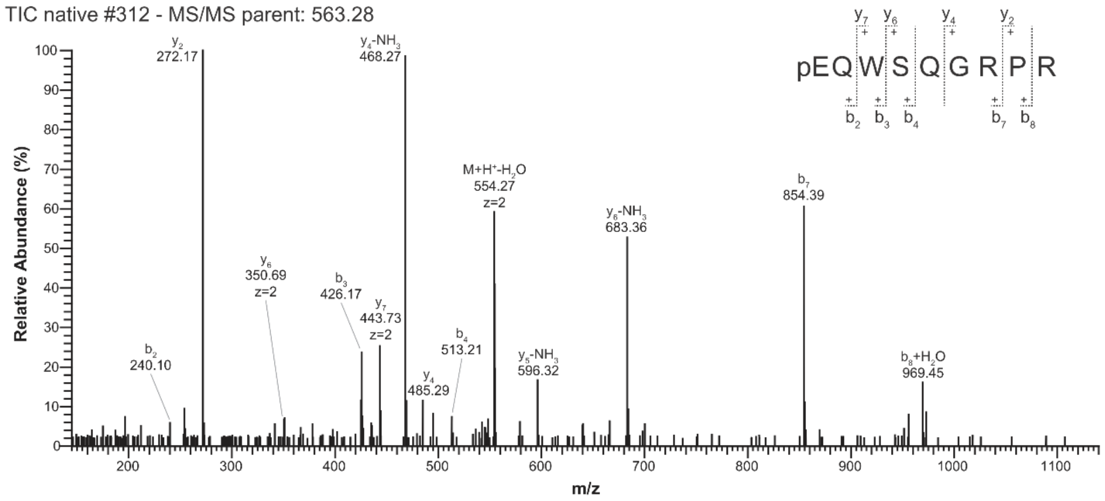

Figure S5. MS2 spectra of pEQWSQGRPR. Representative MS/MS spectra of the *m*/*z* 563.28 (z=2) precursor ion for the top-down annotation of a BPP.

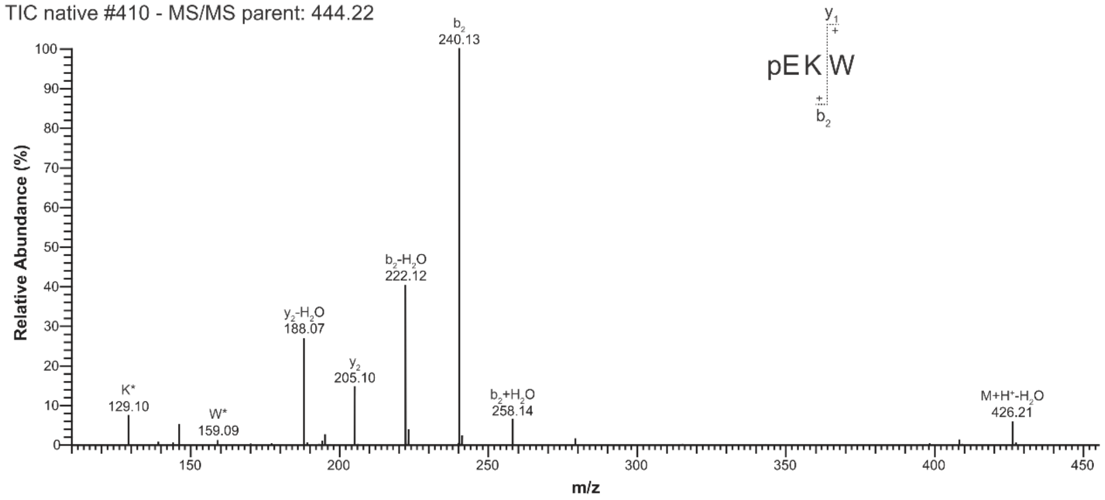

Figure S6. MS2 spectra of pEKW. Representative MS/MS spectra of the *m*/*z* 444.22 precursor ion for the de novo annotation of a small tripeptic svMP inhibitor.

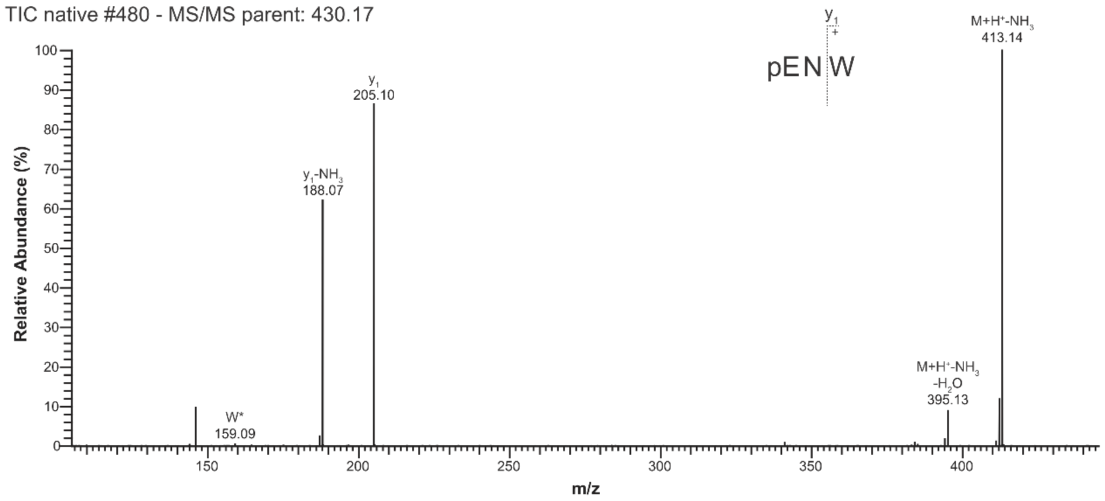

Figure S7. S2 spectra of pENW. Representative MS/MS spectra of the *m*/*z* 430.17 precursor ion for the de novo annotation of a small tripeptic svMP inhibitor.

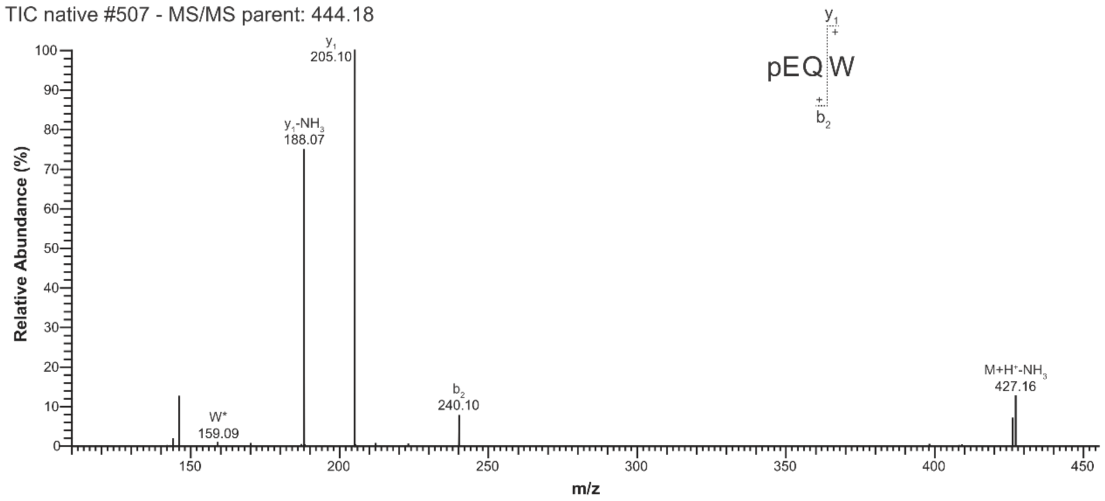

Figure S8. MS2 spectra of pEQW. Representative MS/MS spectra of the *m*/*z* 444.18 precursor ion for the de novo annotation of a small tripeptic svMP inhibitor.

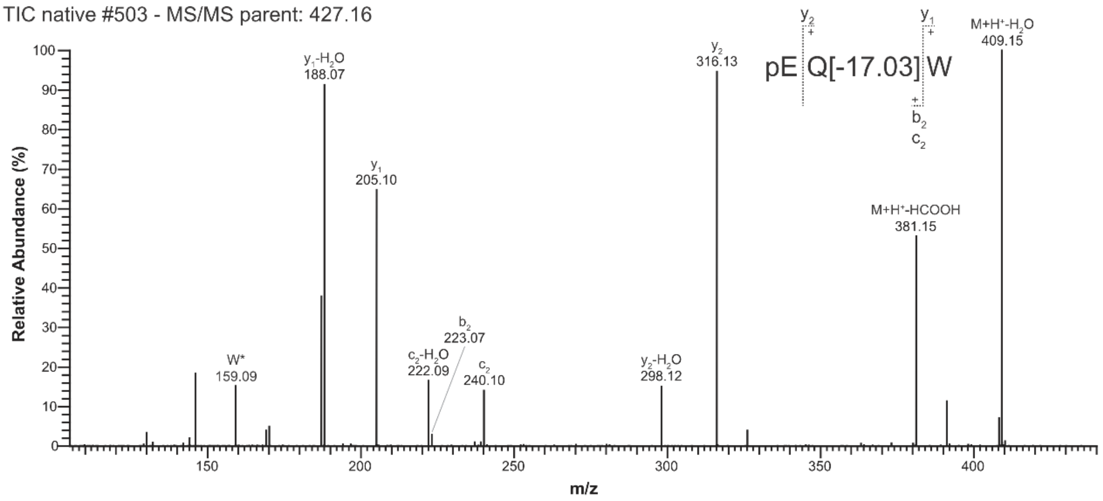

Figure S9. MS2 spectra of pEQ[-17.03]W/pEEW. Representative MS/MS spectra of the *m*/*z* 427.16 precursor ion for the de novo annotation of a small tripeptic svMP inhibitor.

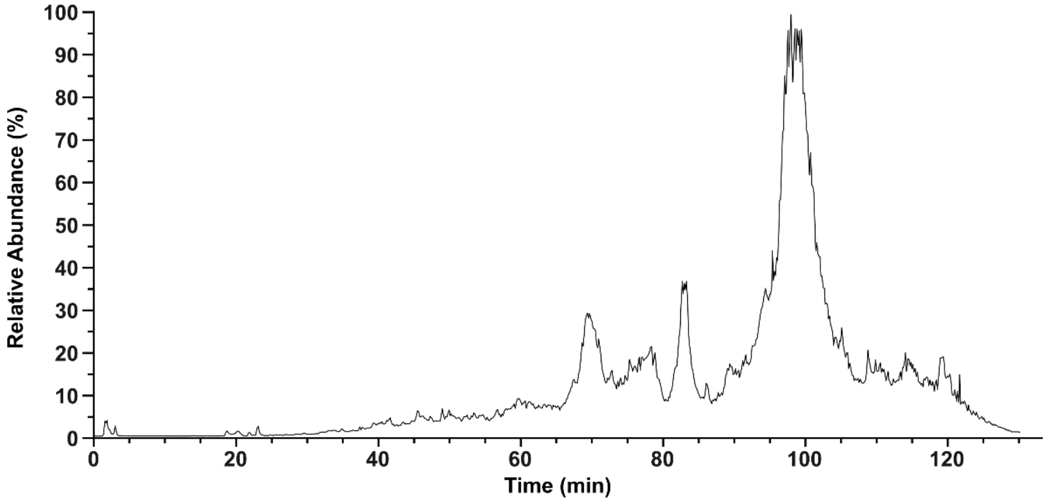

Figure S10. *P. flavoviridis* reduced venom MS TIC for IMP and TD. The total ion counts from *P. flavoviridis* crude venom were measured by an HPLC-ESI-MS of reduced crude venom. The relative abundance was set to 100% for the highest peak.

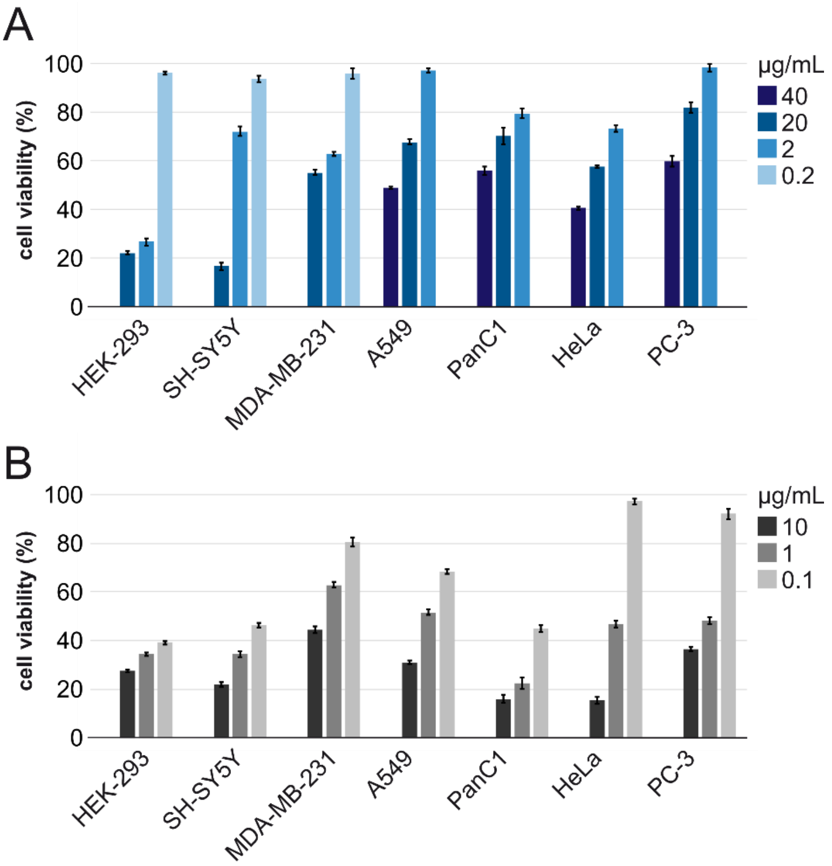

Figure S11. *P. flavoviridis* venom tested for cytotoxicity against different human cell lines. The effect on cell viability (%) of (A) *P. flavoviridis* crude venom was determined at various concentrations (0.2-40.0 µg/mL) and (B) Doxorubicin (0.1-10.0 µg/mL) as control after 48 h treatment by an MTT assay at 570 nm. One non-cancerous (HEK-293) and six cancerous human cell lines were tested. Error mean in ±SD.

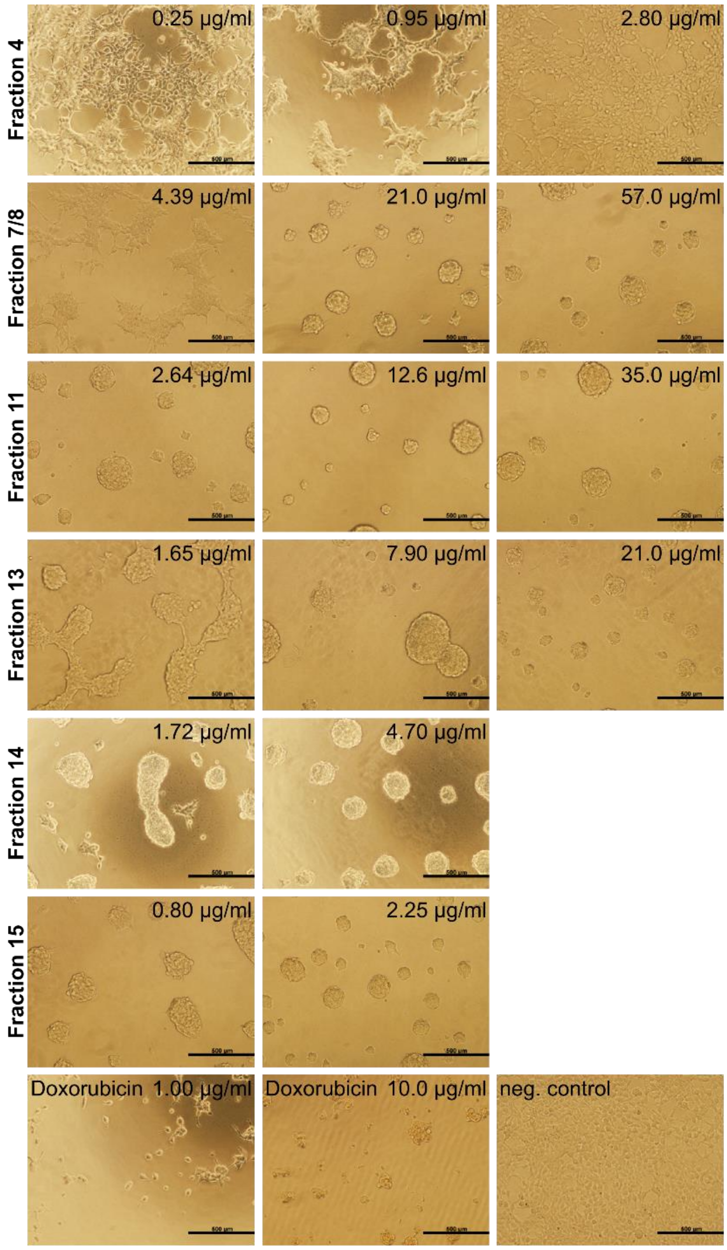

Figure S12. SH-SY5Y cells after 48 h treatment with different *P. flavoviridis* venom fractions. Single RP-HPLC venom fractions of *P. flavoviridis* with the mentioned concentration in µg/mL were tested against human neuroblastoma SH-SY5Y cells. Imaged were taken after 48 h treatment at 37 °C. Doxorubicin was used as positive cytotoxic control drug and no stimulation as negative control.

Table S1. Venom proteins and peptides identified from *Protobothrops flavoviridis*. Assignment of venomic components by crude venom intact mass profiling (IMP, method A), bottom-up (BU, method C) and top-down (TD, method D). Peak numbers are based on the RP-HPLC annotation (Figure 2) and low abundant peaks in the HPLC, but detectable in the IMP, are marked by #. Sequence tags were obtained *de novo* from MS/MS spectra and identified against a non-redundant *Protobothrops flavoviridis* protein database (taxid: 88087) by BLASTP. SDS-PAGE and intact mass profile analysis provided the average molecular weight. Most abundant mass in a IMP TIC are asterisked. Method B with no distinct R_T are marked by °. Monoisotopic TD masses are marked by an indexed m.

Link for the Excel-File
